# Echinococcal hepatic lesion mimicking metastasis from colon cancer: two case reports

**DOI:** 10.1186/s12893-021-01150-1

**Published:** 2021-03-20

**Authors:** Marionna Cathomas, Paolo Abitabile, Rok Dolanc, Christine Glaser, Gieri Cathomas

**Affiliations:** 1 Department of Visceral Surgery and Medicine, Inselspital, Bern University Hospital, 3010, Bern, Switzerland; 2 Sole Chirurgie Parkresort, Rheinfelden, Switzerland; 3 Riva Klinik Rheinfelden, Rheinfelden, Switzerland; 4 Department of Surgery, Cantonal Hospital Baselland, Liestal, Switzerland; 5 Institute of Pathology, Cantonal Hospital Baselland, Liestal, Switzerland

**Keywords:** Echinococcus, Echinococcal cyst, Colorectal cancer, Metastasis, Liver lesion

## Abstract

**Background:**

Echinococcus is a worldwide zoonosis, primarily causing liver lesions. Accidentally detected, these lesions enter the differential diagnosis of a tumor, including metastasis. This situation is especially challenging in patients with colorectal cancer, as both diseases affect mainly the liver.

**Case presentation:**

We report two patients with a newly diagnosed colorectal cancer. Pre- and intraoperatively radiological imaging revealed hepatic lesions which were resected on suspicion of colorectal cancer metastasis. Histology showed granulomatous lesions with characteristic parasitic membrane consistent with an echinococcal cyst. The diagnosis was confirmed by specific polymerase chain reaction.

**Conclusions:**

Focal hypoechoic liver lesion in patients with colorectal cancer should be primarily considered as a liver metastasis and resected whenever feasible. Other uncommon etiologies, including parasitic lesion as echinococcal cysts, should be taken in consideration, as this could lead to major changes of the management and prognosis of the affected patients.

## Background

Echinococcosis is an unusual but clinically relevant infectious disease caused by helminthic parasites. Although the disease can become manifest by local symptoms due to the parasitic cyst, echinococcal lesions often remain asymptomatic for a long time and are accidentally detected by radiological imaging performed for unrelated clinical symptoms [1, 2]. In this situation, the lesions enter the differential diagnosis of a tumorous process. This is especially challenging during tumor staging of patients with colorectal cancer, as the liver is the primary target for both diseases, the echinococcal cyst and the colorectal cancer.

In this report, we present two patients with colorectal cancers with hepatic echinococcal cysts, primarily considered being liver metastasis.

## Case presentation

**Fig. 1:**
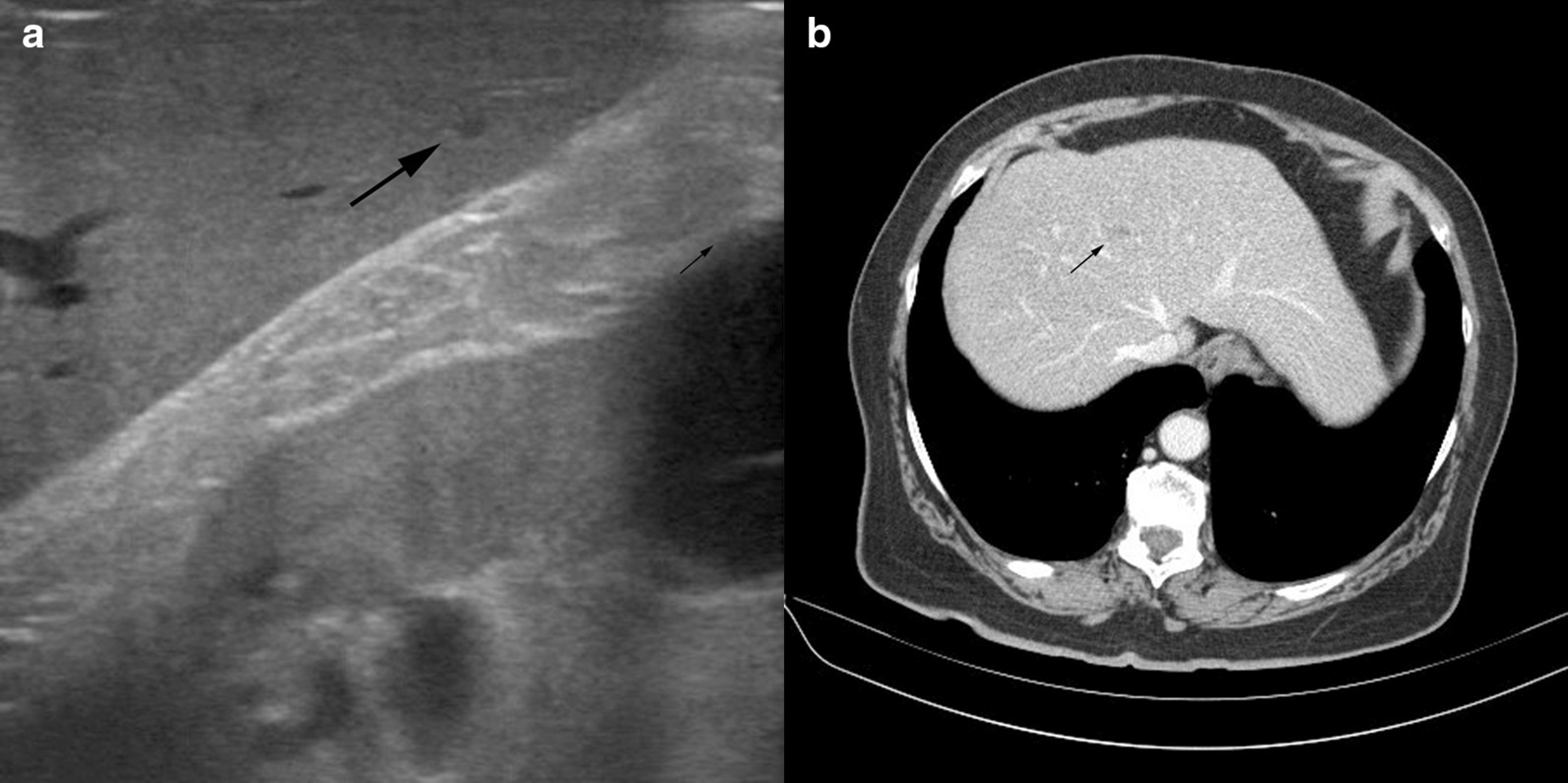
**a** Intraoperative ultrasound of the male patient with a hepatic lesion in segment V. **b** Preoperative computer tomography scan of female patient with a hepatic lesion in segment VI
B. A 76-year-old woman consulted her family doctor for stool irregularities. No further symptoms were reported. A normocytic, normochromic anemia was diagnosed but otherwise, the examination of the family doctor showed no abnormality. A colonoscopy was initiated and a stenosing mass extending from the distal colon to the proximal sigmoid colon were found. After histological confirmation of an adenocarcinoma, preoperative staging by computer tomography showed a focal liver parenchymal change in segment VI with a metastasis-compatible contrast enhancing (Fig. 1b). Intraoperatively, an approximately 6 mm, hyperechogenic structure in segment VI could been visualized by sonography. A left hemicolectomy was performed with an excisional biopsy of the hepatic mass.

A. An 83-year-old man consulted his family doctor due to dyspnea. The dyspnea increased in the last weeks and occurred mainly during physical activity. No other symptoms were reported. Apart from impaired hearing, the patient’s history was uneventful. So far, no colonoscopy was performed. Furthermore, the patient had no previous abdominal surgery. Although he was a retired farmer, he continued to work on his farm and in former years, he regularly went to the forest.
  Physical examination of the patient was inconspicuous. Laboratory analysis revealed a microcytic, hypochromic anemia. For further assessment of the anemia, a colonoscopy was performed and revealed a non-stenosing carcinoma of the ascending colon. No distant metastasis were found by staging computer tomography scan. During primary surgical resection, routine intraoperative ultrasound showed a 10 mm hepatic lesion in segment V (Fig. 1a). Due to a suspected liver metastasis of the colon cancer, resection of the lesion was performed.

**Fig. 2:**
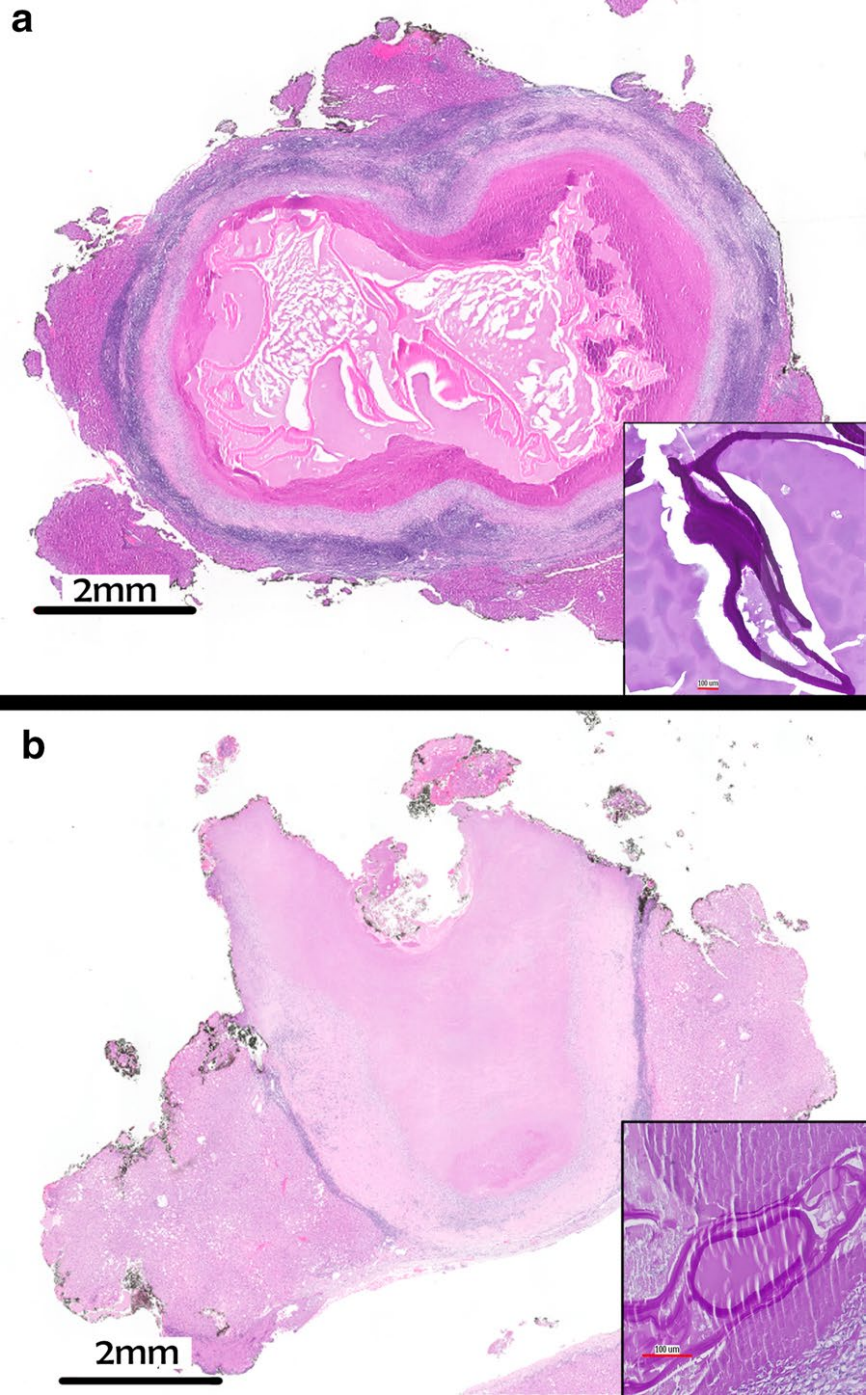
The histology of the liver lesion shows a compact membrane and amorphic cystic content (H&E stain). Insert: Visualization of the characteristic Periodic-Acid-Schiff (PAS)-stain strongly positive membrane of the parasite. **a** male patient; **b** female patient

The pathological workup of both colorectal cancers showed a moderately differentiated pT3 adenocarcinoma with no lymph node metastasis. The examination of the two liver resection specimens revealed a central necrotic cystic lesion with a granulomatous and lymphocytic rim (Fig. 2). Further histochemical staining highlighted the Periodic-Acid-Schiff (PAS)-stain positive germinal layer consistent with echinococcal cysts (Fig. 2, inserts) [3]. Vital parasites (scolices) could not be detected, so that the diagnosis was further confirmed by molecular tests using specific polymerase chain reaction (PCR). These tests revealed *Echinococcus multilocularis* (*E. multilocularis*) and *Echinococcus granulosus* (*E. granulosus*)- DNA in the lesion of the male and female patient, respectively.

Following consultation with the infectious disease specialist, no anti-helminthic treatment was initiated in the male patient due to advanced age, complete resection of the parasitic cyst and lack of vital scolices in the surgical specimen. The female patient was treated with albendazole 400 mg twice per day [14]. Both colon cancers were nodal negative (AJCC/UICC tumor stage II) and therefore no adjuvant chemotherapy was applied. At three years follow-up, both patients were free of cancer and there was no evidence of parasitic disease.

## Discussion and conclusion

In the cases presented, a single pre- and intraoperatively observed hepatic lesion of unknown origin in patients with colorectal cancer were described, which were initially interpreted as a metastatic disease. The histological examination revealed a cystic granulomatous lesion with a characteristic PAS-positive parasitic membrane. Scolices were not detected, however, the parasite is degraded and not visible in more than 50 % of all echinococcal cysts [4]. Therefore, the final diagnosis was confirmed by molecular means, i.e., PCR assay.

Echinococcosis is a worldwide zoonosis encompassing different strains with major epidemiological variations [5]. The incidence of echinococcosis depends on the geographic regions and Europa and Asia are considered as endemic regions [6]. Recently reports showed, however, cases of echinococcosis in non-endemic regions [3, 7]. Urbanization of coyote populations as well as an increase in accidentally infected domestic dogs is assumed to explain this development [8]. Further, globalization and immigration may increase the spread of this parasite [9, 10]. According to the World Health Organization (WHO), alveolar echinococcosis is considered one of the 20 neglected tropical diseases [11]. The absence of a worldwide obligation to report confirmed cases and the lack of physicians’ awareness may lead to underdiagnoses of this emerging disease [9].

For human, mainly two types of Echinococcus are clinically relevant: *E. granulosus* and *E. multilocularis* causing cystic echinococcosis and alveolar echinococcosis, respectively [2]. In the natural cycle of this helminth parasite, the human is an aberrant dead-end host. The incubation time of echinococcosis can be up to 15 years [12]. Due to this long period, the exact transmission way can rarely be elucidated. Furthermore, the clinical symptoms of an infected patient are non-specific, including right upper quadrant pain, malaise or weight loss [2].

There are some major differences of the clinical presentation between *E. multilocularis* and *E. granulosus* [1]. *Echinococcus multilocularis* is mainly restricted to the liver and shows a tumorous, infiltrating growth. In rare cases, the parasite may grow into adjacent structures or even affect distant organs. Based on these characteristics, the WHO has developed a classification specifically for *E. multilocularis*, the so-called PNM stages, analogous to the TNM classification [13]. Due to the aggressive character of *E. multilocularis*, therapy is generally indicated, primarily a surgical resection. Prior to surgery, benzimidazole therapy is administered, which is continued postoperatively. In contrast, *E. granulosus* is usually limited to the liver; however, other organs can be affected including lung, kidney and the brain. A disease with *E. granulosus* is not an absolute treatment indication and a wait and watch strategy may be an acceptable option [14, 15].

In summary, we present two patients with suspected metastatic colorectal cancer finally diagnosed as parasitic cysts. Metastatic colorectal cancer implies tumor stage IV with a 5-year survival of less than 10 %, whereas survival in stage II colorectal cancer is in the range of 85 % [16]. On the other side, little is known about the effect of an accidental cytotoxic chemotherapy on echinococcal disease, but a suppressive effect on the parasite has been described [17]. Finally, any focal hypoechoic liver lesion, even when considered benign, should be resected whenever feasible. Only histological analysis of liver processes in cancer patients can exclude a liver metastasis or detect other lesions, including rare events as the echinococcal cyst described, leading to a major change of the management and prognosis of the affected patients.

## Data Availability

Data sharing is not applicable to this article, as no datasets were generated or analyzed during the current study.

## Abbreviations

AJCC: American Joint Committee on Cancer
*E. multilocularis*: *Echinococcus multilocularis*
*E. granulosus*: *Echinococcus granulosus*
H&E: Hematoxylin and eosin stain
PAS-Stain: Periodic-Acid-Schiff-stain
PCR: Polymerase chain reaction
PNM: P = parasitic mass in the liver, N = involvement of neighbouring organs, and M = metastasis
UICC: Union for International Cancer Control
